# *PPARGC1A* rs8192678 and *NRF1* rs6949152 Polymorphisms Are Associated with Muscle Fiber Composition in Women

**DOI:** 10.3390/genes11091012

**Published:** 2020-08-27

**Authors:** Thomas Yvert, Eri Miyamoto-Mikami, Takuro Tobina, Keisuke Shiose, Ryo Kakigi, Takamasa Tsuzuki, Mizuki Takaragawa, Noriko Ichinoseki-Sekine, Margarita Pérez, Hiroyuki Kobayashi, Hiroaki Tanaka, Hisashi Naito, Noriyuki Fuku

**Affiliations:** 1 Graduate School of Health and Sports Science, Juntendo University, Chiba 270-1695, Japan; thomaspaul.yvert@universidadeuropea.es (T.Y.); eri-miyamoto@juntendo.ac.jp (E.M.-M.); mizuki854@gmail.com (M.T.); noriko.sekine@ouj.ac.jp (N.I.-S.); hnaitou@juntendo.ac.jp (H.N.); 2 Faculty of Sport Sciences, Universidad Europea de Madrid, 28670 Madrid, Spain; margarita.perez@universidadeuropea.es; 3 Faculty of Nursing and Nutrition, University of Nagasaki, Nagasaki 851-2195, Japan; tobitaku@sun.ac.jp; 4 Faculty of Education, University of Miyazaki, Miyazaki 889-2192, Japan; kshiose@cc.miyazaki-u.ac.jp; 5 Faculty of Management & Information Science, Josai International University, Chiba 283-8555, Japan; rkakigi@jiu.ac.jp; 6 Faculty of Pharmacy, Meijo University, Aichi 468-8503, Japan; takamasa425@gmail.com; 7 Faculty of Liberal Arts, The Open University of Japan, Chiba 261-8586, Japan; 8 Department of General Medicine, Mito Medical Center, Tsukuba University Hospital, Ibaraki 310-0015, Japan; hrkoba1@gmail.com; 9 Faculty of Sports and Health Science, Fukuoka University, Fukuoka 814-0180, Japan

**Keywords:** SNP, myosin heavy chain, PGC-1α, nuclear respiratory factor, genotype score

## Abstract

*PPARGC1A* rs8192678 G/A (Gly482Ser) and *NRF1* rs6949152 A/G polymorphisms have been associated with endurance athlete status, endurance performance phenotypes, and certain health-related markers of different pathologies such as metabolic syndrome, diabetes, and dyslipidemia. We hypothesized that they could be considered interesting candidates for explaining inter-individual variations in muscle fiber composition in humans. We aimed to examine possible associations of these polymorphisms with myosin heavy-chain (MHC) isoforms as markers of muscle fiber compositions in vastus lateralis muscle in a population of 214 healthy Japanese subjects, aged between 19 and 79 years. No significant associations were found in men for any measured variables. In contrast, in women, the *PPARGC1A* rs8192678 A/A genotype was significantly associated with a higher proportion of MHC-I (*p* = 0.042) and with a lower proportion of MHC-IIx (*p* = 0.033), and the *NRF1* rs6949152 AA genotype was significantly associated with a higher proportion of MHC-I (*p* = 0.008) and with a lower proportion of MHC IIx (*p* = 0.035). In women, the genotype scores of the modes presenting the most significant results for *PPARGC1A* rs8192678 G/A (Gly482Ser) and *NRF1* rs6949152 A/G polymorphisms were significantly associated with MHC-I (*p* = 0.0007) and MHC IIx (*p* = 0.0016). That is, women with combined *PPARGC1A* A/A and *NRF1* A/A genotypes presented the highest proportion of MHC-I and the lowest proportion of MHC-IIx, in contrast to women with combined *PPARGC1A* GG+GA and *NRF1* AG+GG genotypes, who presented the lowest proportion of MHC-I and the highest proportion of MHC-IIx. Our results suggest possible associations between these polymorphisms (both individually and in combination) and the inter-individual variability observed in muscle fiber composition in women, but not in men.

## 1. Introduction

Human skeletal muscles are composed of different types of myofibers: type I (slow-twitch oxidative), type IIa (fast-twitch oxidative), and type IIx (fast-twitch glycolytic) that differ in morphology, organelle content, energy metabolism, and contractile function [1]. Furthermore, skeletal muscle presents a degree of plasticity: changes in composition occur with age [2] and in response to external stimuli such as exercise training [3]. Indeed, it is well documented that type I fibers have greater aerobic capacity and are more resistant to fatigue than type II fibers [4,5,6]. The contribution of slow-twitch muscle fibers to endurance performance is now well documented. A large number of studies have indicated that athletes in aerobic and endurance-oriented sports present high slow-twitch fiber percentages [6,7,8,9,10]. Further, Bergh et al. [7] found a direct relationship between maximum oxygen uptake (VO_2max_) and the percentage of slow-twitch fibers. In contrast, sedentary individuals and sprinters have a greater proportion of fast-twitch muscle fibers [11]. The frequent use of oxidative muscle fibers that utilize fat as a fuel source to generate ATP leads to a reduction in fat mass and to an improvement in insulin sensitivity [4,5]. Approximately 40% of the variability observed in body fat percentage can be attributed to myofiber composition [12]; a low percentage of type I fibers is considered to be a risk factor for the development of obesity and insulin resistance [13,14], and the proportion of type II fibers is related to blood pressure and hypertension [15].

Skeletal muscle content presents important inter-individual variability in terms of fiber composition: from 15% to 85% for type I fibers, from 5% to 77% for type IIa fibers, and from 0 to 44% for type IIx fibers [16]. This variability seems to be due to an important genetic component; heritability estimates are typically between 45% and 99.5%, depending on the specific study [17,18]. Several genetic polymorphisms have already been found to be related to muscle fiber composition, especially in genes involved in energetic metabolic functions, cytoskeletal functions, or circulatory functions [19]. Peroxisome proliferator-activated receptor γ coactivator-1 α (PGC-1α) is a coactivator of transcription involved in several aspects of skeletal muscle physiology such as mitochondrial biogenesis, glucose utilization, fatty acid oxidation, thermogenesis, gluconeogenesis, and insulin signaling [20]. PGC-1α is responsible for the regulation of lipid and carbohydrate metabolism and for improving muscle fiber oxidative capacity by increasing the number and activity of mitochondria through the upregulation of nuclear respiratory factors NRF1 and NRF2 [21]. These respiratory factors are proposed to upregulate the transcription of several nuclear-encoded respiratory genes [22,23] and to induce the expression of the mitochondrial transcription factor A (TFAM) [21,22]. It should be noted that the PGC-1α–NRF–TFAM pathway contributes to the regulation of exercise-induced changes in muscle fibers towards a more oxidative phenotype [24,25]. PGC-1α has been shown to directly co-activate different myocyte enhancer factor 2 (Mef2) proteins involved in the induction and maintenance of muscle differentiation; this is considered critically important for switching among muscle fiber types or for myofiber transformation to oxidative muscle fibers [25]. NRF1 appears to regulate *Mef2A* gene expression (considered one of the main transcription factors for glucose transporter GLUT4) and activate a cascade that coordinates the expression of respiratory chain components; it also regulates glucose transport in striated muscles [26,27]. Overexpression of PGC-1α in mice reveals oxidative type I fiber dominance [25], while PGC-1α knock-out mice exhibit glycolytic type IId/x and IIb fiber dominance [28]. Nevertheless, mechanisms explaining how the PGC-1α–NRF–TFAM pathway influences these changes in muscle fiber type to contribute to inter-individual variations are equivocal.

There are several genetic polymorphisms already described in genes involved in the PGC-1α–NRF–TFAM pathway. Among these, the *PGC-1α* (*PPARGC1A*) rs8192678 G/A (Gly482Ser) and *NRF1* rs6949152 A/G polymorphisms are candidates that influence mitochondrial function. For example, the *PPARGC1A* rs8192678 G/A polymorphism, causing Gly482Ser amino acid replacement, has been reported to be associated with endurance athlete status in several case–control studies [24,29,30,31] as well as with several cardiorespiratory fitness phenotypes related to endurance performance [30,32]. It has also been linked to certain health-related markers in different pathologies such as dyslipidemia, metabolic syndrome, insulin resistance, and type 2 diabetes [30,33,34,35]. Steinbacher et al. [36] observed that *PPARGC1A* Gly482Ser polymorphism was related to a lack of training-induced increase in slow-contracting oxidative fiber content in 28 males aged between 50 and 69 years. In addition, the *NRF1* rs6949152 polymorphism, located in an intronic region, was reported to involve significant interactions between genotypes and endurance training responses in young men [37]. Further, the *NRF1* locus (7q32) has been shown to present suggestive evidence of linkage with baseline levels of VO_2max_ in healthy sedentary African Americans and with VO_2max_ training responses in Caucasian Americans [38]. However, the functional molecular mechanisms associating these genetic variants with respective pathologic conditions or physical performance related to skeletal muscle tissue are largely unknown.

Taken together, these data suggest *PPARGC1A* and *NRF1* as candidate genes for explaining inter-individual variation in muscle fiber type composition. However, there are no published reports available regarding possible associations between *PPARGC1A* and *NRF1* genotypes and human skeletal muscle content. Therefore, we aimed to examine possible associations involving *PPARGC1A* rs8192678 G/A (Gly482Ser) and *NRF1* rs6949152 A/G polymorphisms and skeletal muscle fiber composition.

## 2. Subjects and Methods

### 2.1. Subjects

In total, 214 healthy non-active Japanese subjects agreed to participate in this study; they were 107 men and 107 women, aged between 19 and 79 years, without any chronic illness or clinical history that could alter muscle composition. The subjects were recruited from Juntendo University and Fukuoka University. Before volunteering, the subjects were given full oral and written information regarding the procedures involved in the study and possible risks associated with participation. All of the subjects signed a written informed consent. The study was performed in accordance to the Helsinki Declaration regarding the conduct of clinical research and was approved by the Ethics Committees of Juntendo University and Fukuoka University.

### 2.2. Genotyping

Total DNA was isolated from venous blood using the QIAamp DNA Blood Mini Kit (Qiagen, Hilden, Germany), according to the manufacturer’s instructions. Total DNA content was measured using a NanoDrop 8000 spectrophotometer (Thermo Fisher Scientific, Waltham, MA, USA). DNA concentration was adjusted to 10 ng/µl with Tris-EDTA buffer, and samples were stored at 4 °C. *PPARGC1A* G/A (Gly482Ser) rs8192678 and *NRF1* rs6949152 A/G polymorphisms were genotyped using a real-time thermocycler (QuantStudio™ 5, Applied Biosystems, Waltham, MA, USA) using TaqMan SNP Genotyping Assays: *PPARGC1A* rs8192678 assay ID: C___1643192_20, *NRF1* rs6949152 assay ID: C__29144830_10. PCR conditions were as previously described [2]. The combination of the two polymorphisms was determined using a genotype score: based on Akaike Information Criteria (AICs) of each genetic model, we attributed a score of 2 to the “higher MHC-I” genotype found for each polymorphism (*PPARGC1A* rs8192678 AA and *NRF1* rs6949152 AA genotypes), whereas a score of 0 was assigned to the “lower MHC-I” genotype (*PPARGC1A* rs8192678 GG+GA and *NRF1* rs6949152 AG+GG genotypes). Then, we summed the scores of the two genotypes.

### 2.3. Muscle Biopsy

**Figure 1:**
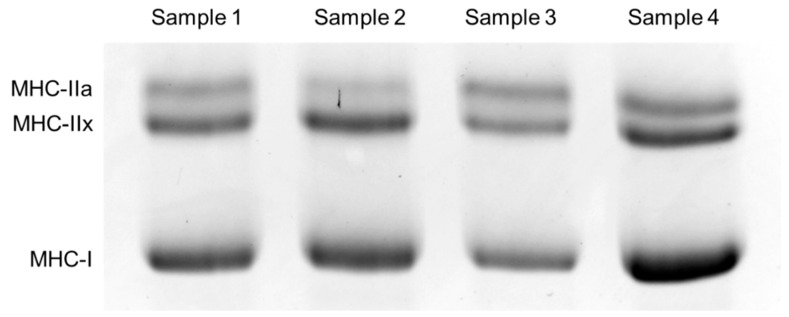
Typical image of myosin heavy-chain (MHC) isoforms determined by SDS-PAGE.

Muscle samples were obtained and analyzed as previously described [39]. Briefly, 10–15 mg of the vastus lateralis muscles were collected using a disposable needle biopsy instrument (Max Core or Magnum; C.R. Bard, Covington, GA, USA). We collected the biopsies from approximately 15 cm above the patella in both legs of each subject under ultrasound imaging (Noblus; Aloka, Tokyo, Japan) and avoided the inclusion of subcutaneous fat and the subfascial and myotendinous parts as far as possible. In addition, any visible non-muscle tissues (e.g., fat tissue) were removed from the biopsy samples. As shown in Figure 1, myosin heavy-chain (MHC) isoform percentages (an indicator of muscle fiber composition) were analyzed by glycerol SDS-PAGE using a calibrated densitometer (Chemi-Doc Touch Imaging System, Bio-Rad Laboratories, Hercules, CA, USA) and analytical software (Image Laboratory software v.5.2.1; Bio-Rad Laboratories, Hercules, CA, USA).

### 2.4. Statistical Analysis

Data are expressed as means ± SD. Chi-square analysis was used to confirm whether the observed genotype frequencies were in Hardy–Weinberg equilibrium. The possible association of the *PPARGC1A* rs8192678 G/A (Gly482Ser) and *NRF1* rs6949152 A/G genotypes was analyzed using multiple linear regression analysis. As previously reported [2], the age of the subjects correlated significantly with the proportion of MHC isoforms among men and women, therefore our analysis included age as a covariant. AICs were calculated for each genetic model to determine the best-fitting genetic model (additive, dominant, and recessive). For the analysis of the combined effects of the two polymorphisms, a genotype score was calculated using the modes of inheritance presenting the best-fitting model for each polymorphism, based on AIC. The genotype score for the combined effects of *PPARGC1A* rs8192678 G/A (Gly482Ser) and *NRF1* rs6949152 A/G polymorphisms was analyzed using multiple linear regression. Statistical significance was set at *p* < 0.05. Statistical analyses were performed using JMP Pro v.12 software (SAS Institute, Cary, NC, USA).

## 3. Results

The genotyping success ratio was 210/214 (98.1%) for the *PPARGC1A* rs8192678 G/A (Gly482Ser) and the *NRF1* rs6949152 A/G polymorphisms. Both polymorphisms were within the Hardy–Weinberg equilibrium (*p* = 0.970 and *p* = 0.176, respectively). The main characteristics of the study participants are presented in Table 1. Muscle fiber compositions were significantly different between genders, with women presenting higher percentages of MHC-I and lower percentages of MHC-IIa and MHC-IIx, compared to men.

No significant associations were found in men between genotypes and fiber type distribution for both the *PPARGC1A* rs8192678 G/A (Gly482Ser) and the *NRF1* rs6949152 A/G polymorphisms. In contrast, in women, the *PPARGC1A* rs8192678 G/A polymorphism was significantly associated with MHC-I for the additive genetic model and with MHC-IIx for the dominant genetic model. For *NRF1* rs6949152 A/G, a significant association was observed for MHC-I when considering the additive and recessive genetic models and with MHC-IIx when considering the recessive genetic model, as shown in Table 2.

**Figure 2:**
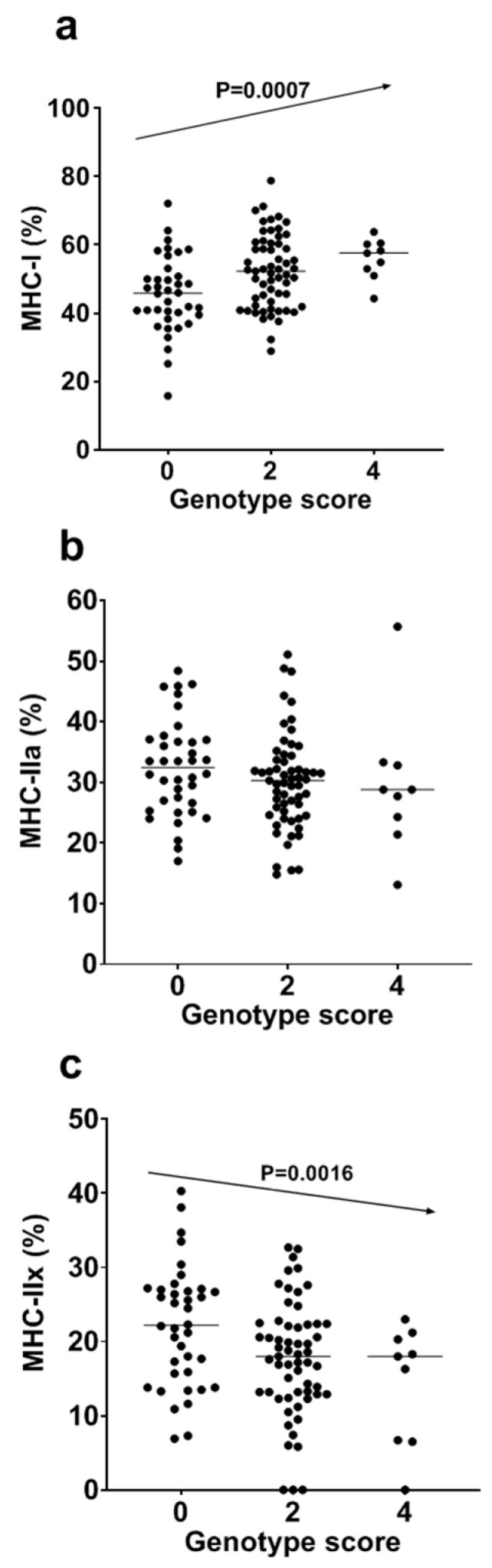
Genotype scores as combined effects of *PPARGC1A* rs8192678 G/A (Gly482Ser) (AA = 2 vs. GG + GA = 0) and *NRF1* rs6949152 A/G (AA = 2 vs. AG + GG = 0) polymorphisms on MHC-I (**a**), MHC-IIa (**b**), and MHC-IIx content (**c**) in women.

As presented in Figure 2 and Table 3, the genotype scores of the modes presenting the most significant results for *PPARGC1A* rs8192678 G/A (Gly482Ser) and *NRF1* rs6949152 A/G polymorphisms were significantly associated with MHC-I (*p* = 0.0007; Figure 2a) and MHC IIx (*p* = 0.0016; Figure 2c) but not with MHC-IIa (*p* = 0.1657; Figure 2b) in women. In women, an increase of 1 point in the genotype score was associated with a 2.94-time higher percentage of MHC-I (2.82 times higher when adjusted by age) and with a 2.02-time lower percentage of MHC-IIx (1.99 times lower when adjusted by age) (Table 3).

## 4. Discussion

To our knowledge, this is the first attempt to determine a possible association between *PPARGC1A* and *NRF1* polymorphisms and human skeletal muscle fiber composition, considering sex-specific differences, in a relatively large sample. Both *PPARGC1A* rs8192678 G/A (Gly482Ser) and *NRF1* rs6949152 A/G polymorphisms were individually associated with muscle fiber composition in women, but not in men, also when combined together by genotype scores. Thus, the *PPARGC1A* rs8192678 and *NRF1* rs6949152 polymorphisms seem to influence, at least partly, phenotypic traits related to inter-individual variation in skeletal muscle fiber composition in women.

In the present study, our results showed increased oxidative-oriented proportions of muscle fibers in *PPARGC1A* 482 AA (Ser/Ser) genotype carriers compared to GG (Gly/Gly) or GA (Gly/Ser) carriers and in *NRF1* AA genotype carriers compared to GG or AG carriers. For the *NRF1* rs6949152 polymorphism, this result is in accordance with previous literature, the AA genotype having already been found to be associated with training responses in terms of aerobic capacity, with significantly higher ventilatory thresholds after 18 weeks of endurance training in 102 young Chinese men [40]. However, in the case of *PPARGC1A* rs8192678 Gly482Ser, our results are more controversial. In fact, in a Caucasian population, in contrast to our results, the *PPARGC1A* rs8192678 482Ser allele has been observed to be less frequent in endurance athletes than in untrained individuals [24,29,30,31]. Furthermore, carriers of the 482Ser allele present reduced cardiorespiratory fitness and higher risks for metabolic syndrome and type 2 diabetes [30,33,35]. In their study in a reduced sample of Caucasian men (*n* = 28) comparing the *PPARGC1A* rs8192678 Gly482Ser genotype with muscle content trainability, Steinbacher et al. [36] found the Ser allele to be related to a lack of training–induced increase in the proportion of slow-contracting oxidative fibers after 10 weeks of endurance training. Nevertheless, they also observed in the baseline characteristics of their subjects that Ser allele carriers presented significantly higher proportions of slow fibers, which is in accord with our results. In regard to the results obtained for our Japanese cohort, an interesting controversy already exists in the literature concerning Asian populations. In two studies conducted in Chinese and Japanese people, the *PPARGC1A* rs8192678 polymorphism did not exhibit any significant association with higher aerobic capacity or athlete status [37,41], but a non-significant tendency indicated a possible higher frequency of the Ser/Ser genotype in Japanese endurance athletes, especially in those competing at an international level [41]. Even more interesting, in accordance with our results and in contrast to those obtained in Caucasians, Nishida et al. observed an association between the 482Ser allele and higher lactate thresholds in 112 Japanese middle-aged men [32]. These results, taken together, seem to indicate possible ethnic differences in the functional effects of the *PPARGC1A* rs8192678 polymorphism on physical performance related to skeletal muscle activity.

The notable difference in our results between men and women can be explained by several factors such as gender differences in muscle fiber composition and fiber conversion with age or hormonal activity. Several previous studies have found differences between men and women in terms of muscle fiber composition [2,16,42,43], with women presenting higher percentages of slow-twitch fibers than men. Interestingly, this ratio seems to increase with age due to different muscle fiber transitions between the genders: the number of type I fibers decreases with age in men, whereas it increases in women [43]. Important differences in myofiber distribution between genders could be related to sexual hormones. Mitochondria play an essential role in sex steroid hormone biosynthesis and, in turn, sex steroid hormones such as estrogen, progesterone, and testosterone may regulate mitochondrial function [44]. In addition to their presence in the nuclei and plasma membrane, estrogen and androgen are also localized in the mitochondria of a number of cell types and tissues [45]. In mitochondria, estrogens are bound to mitochondrial DNA (mtDNA), suggesting hormonal action on mtDNA transcription and replication. Estrogens can increase the expression of mitochondrial proteins from both nuclear and mitochondrial genomes and favor mitochondrial biogenesis [45,46]. Furthermore, compared with males, the gastrocnemius muscle of female rats exhibits higher mtDNA and protein contents, TFAM protein levels, oxidative and phosphorylative machinery activity, and glutathione peroxidase activity [47]. The regulation of mitochondrial function and biogenesis by estrogens has been extensively reviewed [45,46]. Estrogens increase the expression of PGC-1α and of its downstream targets [48]. In turn, PGC-1α is a co-activator of estrogen-dependent transcriptional activity [49]. It is worth mentioning that Mattingly et al. reported the presence of an estrogen-responsive element (ERE) in the promoter of the *NRF1* gene and observed that knockdown of *NRF1* blocked estradiol stimulation of mitochondrial biogenesis and activity, indicating a mechanism by which estrogens regulate mitochondrial function by increasing NRF1 expression [50]. We can thus speculate that variations of the two studied genetic polymorphisms could have functional impacts on responses of the PGC-1α–NFR–TFAM pathway to sexual hormone signals. For example, we can hypothesize that the *PPARGC1A* rs8192678 polymorphism could affect the capacity of estrogen to activate PGC-1α pathway expression, as well as the reciprocal capacity of PGC-1α to activate estrogen-stimulated transcriptional activity. In addition, we could propose that the *NRF1* rs6949152 polymorphism may affect the functional capacity of its gene promotor ERE, modulating estrogen regulatory effects on its expression.

PGC-1α and NRF1 are generally considered the principal actors in the PGC-1α–NFR–TFAM mitochondrial biogenesis pathway and thus play essential roles in muscle fiber adaptation to different external stimuli. In muscle cells, PGC-1α co-activates the transcriptional functions of NRF1 and NRF2 on the TFAM promoter, thereby increasing TFAM expression [21], especially if activated by exercise [51]. In addition to TFAM, the PGC-1α–NRF pathway also upregulates the mitochondrial transcription factors B1 (TFB1M) and B2 (TFB2M), which are essential components of the mtDNA transcriptional machinery [52]. Collectively, these results support the involvement of the PGC-1α–NRF–TFAM pathway in the coordinated induction of nuclear- and mitochondrial-encoded gene transcription, leading to mitochondrial biogenesis. The PGC-1α pathway is considered to play important physiological roles in regulating fiber specification. Indeed, PGC-1α is preferentially expressed in muscles rich in type I fibers [25]. After chronic exercise, elevated PGC-1α expression has been observed in muscles rich in type II fibers, leading to changes in muscle morphology and gene expression and function [25,53]. Most importantly, muscle-specific PGC-1α transgenic mice present muscle fibers with increased fatigue resistance and exhibit improved endurance exercise performance [25,54]. Additionally, glucose uptake and glycogen synthesis are increased by PGC-1α in muscle, whereas glycolysis is inhibited [53,55]. Moreover, it has been shown in cultured muscle cells that the PGC-1α pathway activates the transcription of fiber-type-specific gene promoters in cooperation with Mef2 protein and serves as a target for calcineurin signaling, which has been implicated in slow-fiber gene expression [25]. This indicates that the PGC-1α pathway plays a key role in the functional conversion of myofibers towards a more oxidative state. Thus, the combined effects of *PPARGC1A* rs8192678 G/A (Gly482Ser) and *NRF1* rs6949152 A/G polymorphisms play an important role in determining muscle fiber composition, because these polymorphisms are located in genes involved in the PGC-1α pathway.

Finally, this study has several limitations, principally related to the reduced sample size, especially because the sample was divided according to gender; there is also a lack of measurements of other parameters related to muscle characteristics, such as the number and cross-sectional area of each fiber. In addition, the method used for MHC isoform analysis may also have certain limitations if used as a single method (e.g., hybrid fiber type analysis [56,57]). Extensive studies are necessary to gather conclusive evidence.

In conclusion, we have identified significant associations between the A (Ser) allele of the *PPARGC1A* rs8192678 G/A (Gly482Ser) polymorphism and the A allele of the *NRF1* rs6949152 A/G polymorphism (both individually and in combination) with a more oxidative-oriented muscular fiber type composition in Japanese women, but not in men. These results are consistent with other studies conducted in Asian populations, analyzing other endurance performance phenotypes. The novelty of this study comes from the molecular genotyping analysis of these two important genes related to inter-individual variation in human muscle fiber type proportions. Further investigations are needed to corroborate our results in Asian populations, as well as in other ethnic groups, in order to better understand the genetic factors that influence human muscular functions and adaptations to the environment and to the different health conditions related to this biological tissue.

## Figures and Tables

**Table 1:** Characteristics of the subjects.

	Men (*n* = 107)	Women (*n* = 107)
Age (years)	47.5 ± 17.9	48.3 ± 16.3
Height (cm)	169.7 ± 5.9	156.7 ± 5.9 ***
Weight (kg)	74.7 ± 11.2	62.5 ± 9.9 ***
BMI (kg/m^2^)	25.9 ± 3.8	25.5 ± 4.3
MHC-I (%)	40.7 ± 11.5	50.3 ± 11.2 ***
MHC-IIa (%)	35.9 ± 8.2	30.9 ± 8.2 ***
MHC-IIx (%)	23.4 ± 9.1	18.8 ± 8.3 ***

Data are expressed as means ± SD. BMI: body mass index, MHC: myosin heavy chain; *** *p* < 0.001.

**Table 2:** Associations of *PPARGC1A* rs8192678 G/A (Gly482Ser) and *NRF1* rs6949152 A/G polymorphisms with MHC-I, -IIa, and -IIx content in men and women.

Gene Name (rs Number)	Genotype			*p* (AIC)		
*PPARGC1A* (rs8192678)				Additive	Dominant	Recessive
Men	GG (*n* = 31)	GA (*n* = 51)	AA (*n* = 22)	GG vs. GA vs. AA	GG + GA vs. AA	GG vs. GA + AA
MHC-I (%)	40.3 ± 10.7	41.6 ± 10.8	39.3 ± 14.6	0.854 (795.8)	0.985 (795.9)	0.764 (795.8)
MHC-IIa (%)	37.5 ± 7.3	34.4 ± 7.2	36.9 ± 11.0	0.366 (724.2)	0.965 (725.1)	0.150 (722.9)
MHC-IIx (%)	22.2 ± 8.9	24.0 ± 9.0	23.8 ± 10.0	0.589 (759.0)	0.981 (759.3)	0.392 (758.6)
Women	(*n* = 36)	(*n* = 52)	(*n* = 18)			
MHC-I (%)	47.7 ± 12.1	50.3 ± 11.0	54.4 ± 7.7	0.042 (809.1) *	0.084 (810.3)	0.112 (810.7)
MHC-IIa (%)	32.3 ± 8.3	30.1 ± 8.0	30.4 ± 9.0	0.368 (750.3)	0.854 (751.1)	0.241 (749.7)
MHC-IIx (%)	20.1 ± 8.9	19.6 ± 7.4	15.2 ± 8.0	0.072 (749.2)	0.033 (747.8) *	0.348 (751.7)
*NRF1* (rs6949152)				Additive	Dominant	Recessive
Men	AA (*n* = 77)	AG (*n* = 21)	GG (*n* = 6)	AA vs. AG vs. GG	AA + AG vs. GG	AA vs. AG + GG
MHC-I (%)	39.8 ± 11.4	44.3 ± 11.6	40.2 ± 13.3	0.334 (794.9)	0.713 (795.7)	0.142 (793.6)
MHC-IIa (%)	36.0 ± 8.5	34.8 ± 7.1	38.0 ± 9.2	0.853 (725.0)	0.341 (724.1)	0.794 (725.0)
MHC-IIx (%)	24.2 ± 9.1	20.9 ± 9.3	21.7 ± 8.3	0.188 (757.6)	0.713 (759.2)	0.125 (756.9)
Women	(*n* = 59)	(*n* = 41)	(*n* = 6)			
MHC-I (%)	52.7 ± 10.5	46.3 ± 11.5	51.0 ± 6.6	0.033 (808.7) *	0.891 (813.3)	0.008 (806.0) **
MHC-IIa (%)	29.8 ± 8.7	32.4 ± 7.4	31.2 ± 8.7	0.221 (749.6)	0.886 (751.1)	0.155 (749.0)
MHC-IIx (%)	17.5 ± 7.7	21.3 ± 8.7	17.8 ± 5.1	0.108 (749.9)	0.745 (752.5)	0.035 (748.0) *

Data are expressed as means ± SD; * *p* < 0.05, ** *p* < 0.01; *p* values show results adjusted by age.

**Table 3:** Association of genotype scores with MHC-I, IIa, and IIx content in women.

−	Model	R^2^ (Adjusted R^2^)	β for GS (95% CI), *p* Value
MHC-I	1	0.104 (0.096)	2.94 (1.26–4.61), *p* = 0.0007
	2	0.140 (0.123)	2.82 * (1.17–4.47), *p* = 0.0010
MHC-IIa	1	0.018 (0.009)	−0.92 (−2.22−0.38), *p* = 0.1657
	2	0.053 (0.035)	−0.83 * (−0.19−0.00), *p* = 0.2014
MHC-IIx	1	0.091 (0.083)	−2.02 (−3.26–(−0.78)), *p* = 0.0016
	2	0.096 (0.078)	−1.99 * (−3.24–(−0.75)), *p* = 0.0020

Model 1: genotype score, Model 2: genotype score and age. GS: genotype score, CI: confidence interval; * age-adjusted regression coefficients for GS.
